# Evaluation of Pre-emptive Analgesia in Total Knee Arthroplasty During Early Post-operative Periods

**DOI:** 10.7759/cureus.41433

**Published:** 2023-07-06

**Authors:** Ajay C Ssamy, Bishnu P Patro, Madhan Jeyaraman, Gurudip Das, Arulkumar Nallakumarasamy, Sankalp Yadav

**Affiliations:** 1 Orthopaedics, All India Institute of Medical Sciences, Bhubaneswar, Bhubaneswar, IND; 2 Orthopaedics, ACS Medical College and Hospital, Dr. MGR Educational and Research Institute, Chennai, IND; 3 Medicine, Shri Madan Lal Khurana Chest Clinic, New Delhi, IND

**Keywords:** total knee arthroplasty, pregabalin, etoricoxib, pre-emptive analgesia, pre-emptive

## Abstract

Introduction: Pre-emptive analgesia is expected to decrease post-operative pain. The degree of soft tissue release is directly related to preoperative deformity; we presume the severity of pain has a similar correlation in patients undergoing total knee arthroplasty (TKA). The main purpose of this research was to evaluate the effects of pre-emptive analgesia of different drugs in TKA with different degrees of preoperative genu varus.

Methods: In this prospective observational study, 67 patients were enrolled with different degrees of genu varus deformity. They were subdivided into two groups: those with ≥15° and those with <15° varus deformities of the knee. Etoricoxib 60 mg and pregabalin 75 mg were administered orally in all the patients as pre-emptive analgesia two hours before surgery. Parameters such as the amount of soft tissue release, visual analog score (VAS), knee range of motion, complications, etc. were documented from the pre-operative period to 72 hours post-TKA.

Results: With pre-emptive analgesia in post-TKA patients, the VAS score demonstrated a statistically significant difference at 24, 48, and 72 hours. The comparison of intraoperative flexion between <15° and ≥15° showed a statistically significant difference with pre-emptive analgesia in post-TKA patients.

Conclusion: The use of etoricoxib 60 mg and pregabalin 75 mg, two hours before surgery reduced the pain scores in patients undergoing TKA with different degrees of genu varus and correlated with intraoperative parameters associated with soft medial tissue release for genu varus.

## Introduction

Osteoarthritis (OA) of the knee involves a gradual loss of cartilage and decreased number of chondrocytes leading to the remodeling of the subchondral bone and the development of new blood vessels. These blood vessels supply osteoblasts and other chemokines that further promotes cartilage erosion and triggers inflammation [1,2]. The mainstay management of early OA knee is using non-steroidal anti-inflammatory drugs (NSAIDs). NSAIDs are associated with severe side effects like hypertension, gastric erosions, upper gastrointestinal bleeding with melena, dyspepsia, etc. [3,4]. So the present trend is to treat OA patients with low-dose NSAIDs and adjuvant therapy like intra-articular hyaluronic acid, corticosteroids, platelet-rich plasma (PRP), mesenchymal stromal cell injections, etc. Recent studies have documented the role of adjuvant therapy being limited to early OA and found that surgical interventions have better outcomes than conventional management in terms of high-grade OA knee [5,6]. The goals of surgery are to reduce pain, decrease disability and improve life quality. Surgical treatment is preferred when conservative management fails, and patient symptoms are not relieved. Total knee arthroplasty (TKA) involves correction of deformity, balancing the collateral ligaments, and arthroplasty of damaged articular cartilage with metal implants. Following TKA, the objective is to provide a pain-free post-operative period and avoid any type of complications, especially post-operative nausea, over-sedation, etc. Neuraxial anesthesia is safer than general anesthesia. Regional anesthesia has been advocated recently as a technique for enhancing post-operative control of pain. Both femoral and sciatic nerve blocks have been used and are connected with a blockade of motor that is prolonged, interferes with early mobilization, and places several patients at risk of falling [7]. Regional anesthesia becomes effective when continued post-operatively because of the long transmission of stimuli from receptors of afferent pain [8-11].

The role of pre-emptive analgesia in decreasing post-operative pain with different degrees of soft tissue release and variable bone cut in TKA is assumed to be different. As the amount of pain following extensive soft tissue release in a severely deformed knee following TKA is more compared to lesser soft tissue release, this study aimed to evaluate the pre-emptive analgesic effect of different drugs in different degrees of soft tissue release following TKA.

We evaluated the pre-emptive role of analgesia in pain reduction in the early post-operative period among patients undergoing total knee arthroplasty. The aim of this study was to find out if there was any correlation between the severity of deformity (amount of soft tissue release) and early post-operative pain in patients who received pre-emptive analgesia.

## Materials and methods

A prospective observational study was conducted after obtaining institutional ethical clearance (IEC/AIIMS BBSR/PG Thesis/2020-21/61, dated November 2, 2020). The indication for surgery was severe degenerative arthritis that had failed adequate non-operative management. Patients younger than 55 years of age or older than 80 years, those with American Society of Anaesthesiologists (ASA) grade >3, history of liver failure, renal or heart-related disease, previous use of drugs that are anti-neuropathic, and patients with neuropathic pain were excluded from the study. Patients with OA knee, primary and secondary with or without fixed flexion deformity (FFD) operated at the Department of Orthopaedics, All India Institute of Medical Sciences, Bhubaneswar, from July 2020 to July 2022, by a senior surgeon were included in the study. A total of 67 patients were recruited after taking written and informed consent. The demography of patients, which includes age, sex, date of surgery, and admission number, was entered in a predesigned proforma.

The routine pre-operative systemic and local examination was conducted for all patients included in the study. Radiological investigations like plain knee radiographs (antero-posterior and lateral view) in standing weight-bearing positions and long-length standing radiographs of lower limbs that were bilateral were taken to assess the degree of deformity of the knee. Each case was subdivided as per the Kellgren and Lawrence system of radiographic grading, and the degree of the deformity was evaluated.

Pre-operative knee pain was assessed using the visual analog score (VAS) with a range of 10 points. The VAS score was evaluated at two hours pre-operatively, and at 2, 6, 24, 48, and 72 hours post-surgery.

All participants in the study received spinal anesthesia; they received 2.5 ml of 0.5% hyperbaric bupivacaine intrathecally. All patients underwent routine primary jig-based total knee arthroplasty using medial parapatellar approach using a posterior stabilized cemented implant with soft tissue releases carried out sequentially depending on the severity of the derformity aiming to achieve equal gaps in both flexion and extension [12].

All patients were managed with a similar peri-operative regimen that included intravenous antibiotics and intravenous analgesia for the initial three days, followed by oral analgesia for another three to five days as needed. For the first 24 hours after surgery, intravenous (IV) paracetamol (1 g) was given every eight hours, followed by a transition to oral celecoxib (200 mg) twice a day, oral paracetamol (1 g) three times a day, and pregabalain (75 mg) once a day until discharge. Breakthrough pain was managed with a stat dose of IV tramadol 50 mg. Adequate prophylaxis against thrombosis was advocated in all patients. Patients were encouraged to start knee range of motion, quadriceps and hamstring strengthening exercises, and ankle pump from day 1 of surgery following weaning of anesthesia. Besides, they were allowed to bear weight while walking from day 2 of surgery with a knee brace and walker.

The outcome assessment of the VAS score was done immediately two hours after the effect of anesthesia weaned off. It varied with each patient, i.e. three to six hours post-operatively. The VAS score was measured at 2, 6, 24, 48, and 72 hours post-operatively. A post-operative plain radiograph was taken to look for implant positioning. General health condition was assessed regularly. The dressing was done on postoperative days 2 and 4. There was no sign of surgical site infection in any study participant. The length of stay varied for each patient from five to seven days.

Statistical analysis

The data was entered into Microsoft Excel 2020 (Microsoft, Redmond, WA) and analyzed using SPSS Statistics, version 26.0 (IBM Corp., Armonk, NY). Continuous variables were presented as means and standard deviations. The categorical variables were presented as proportions or percentages. The association for the dependent and independent variables was calculated by the chi-square test and unpaired t-test for categorical and continuous variables, respectively.

## Results

The baseline demographic characteristics and clinical parameters in terms of mean age, gender, diagnosis, and pre-operative genu varus were identified after obtaining a long-length scannogram. A total of 67 patients were recruited for the study who underwent TKA; these patients completed follow-ups and were analyzed. Seventeen were males and 50 were females; the mean age of patients was 60.16 ​​± 7.04 years, the mean height was 164.78 ± 5.24 cm, and the mean weight was 73.75 ± 7.26 kg. The baseline characteristic summary of the patients is presented in Table 1.

**Table 1:** Demography of the study population

Parameters		Frequency	Percentage
Gender	Female	50	74.6
	Male	17	25.4
Side	Left	36	53.7
	Right	31	46.3
Diagnosis	Osteoarthritis knee	67	100.0
Varus	<15°	20	29.9
	≥15°	47	70.1

Patients were divided into groups of two based on the long-length scannogram: patients with genu varus <15° and the other group with patients with genu varus ≥15° undergoing TKA at our institution (Table 2).

**Table 2:** Varying degree of varus

Total sample		Varus degree		P-value
		<15° (n=20)	≥15° (n=47)	
Sex	Female	15	35	0.610
	Male	5	12	
Side	Left	9	27	0.350
	Right	11	20	

There were 15 females and 5 males in the <15° group and 35 females and 12 males in the ≥15° genu varus group. Both groups had no statistically significant difference. The sequential change in the VAS score among study participants is tabulated in Table 3.

**Table 3:** Changes in the VAS score VAS, visual analog score

Timescale	<15°	≥15°	P-value
VAS at 2 hours before surgery	5.93 ± 0.64	6.00 ± 0.64	0.708
VAS at 2 hours post-surgery	7.89 ± 0.87	8.10 ± 0.78	0.363
VAS at 6 hours post-surgery	7.33 ± 0.81	7.35 ± 0.74	0.911
VAS at 24 hours post-surgery	5.43 ± 1.44	6.40 ± 1.53	0.017
VAS at 48 hours post-surgery	4.11 ± 1.49	5.50 ± 1.14	<0.001
VAS at 72 hours post-surgery	3.09 ± 1.05	3.85 ± 1.30	0.014

When comparing intraoperative parameters for genu varus <15° and ≥15° groups, no statistical difference was seen with respect to gender, side of the surgery, use of screw for the management of the defect, or use of stem extender; only one patient sustained an intraoperative fracture in the tibial articular margin in the less than 15° group as shown in Table 4.

**Table 4:** Comparison of intraoperative parameters for the genu varus <15° and genu varus ≥15° groups MCL, medial collateral ligament

Parameters		Varus ≥15°	Varus <15°	P-value
Gender	Male	12 (25.5)	5 (25.0)	0.963
	Female	35 (74.5)	15 (75.0)	
Side	Left	27 (57.4)	9 (45.0)	0.350
	Right	20 (42.6)	11 (55.0)	
Management of defects (screw and cement)	Yes	9 (19.1)	2 (10.0)	0.355
	No	38 (80.9)	18 (90.0)	
Use of stem	Yes	1 (21.1)	1 (5.0)	0.511
	No	46 (97.9)	19 (95.0)	
Any fracture	Yes	1 (2.1)	0 (0.0)	1.00
	No	46 (97.9)	20 (100.0)	
Deep MCL	Yes	6 (12.8)	5 (25.0)	0.216
	No	41 (87.2)	15 (75.0)	
Superficial MCL	Yes	12 (25.5)	6 (30.0)	0.706
	No	35 (74.5)	14 (70.0)	
Capsule on the medial aspect	Yes	2 (4.3)	5 (25.0)	0.011
	No	45 (95.7)	15 (75.0)	
Capsule on the posterior aspect	Yes	3 (15.0)	8 (17.0)	0.838
	No	39 (83.0)	17 (85.0)	
Osteophytes on the posterior aspect of the femur	Yes	4 (20.0)	18 (38.3)	0.144
	No	29 (61.7)	16 (80.0)	
Hamstring	Yes	47 (100.0)	20 (100.0)	
	No	0 (0.0)	0 (0.0)	
Pie crusting of the MCL	Yes	2 (10.0)	4 (8.5)	0.845
	No	43 (91.5)	18 (90.0)	
Rectus sniff	Yes	0 (0.0)	0 (0.0)	
	No	47 (100.0)	20 (100.0)	
Avulsion of patellar tendon	Yes	0 (0.0)	0 (0.0)	
	No	47 (100.0)	20 (100.0)	

The degree of medial release of the deep medial collateral ligament (MCL) and superficial MCL was not statistically significant in either of the groups. There was a higher release of the medial capsule in the ≥15° varus group, which was found to be statistically significant compared to the <15° of genu varus. In the <15° genu varus group, eight patients received resection of the capsule on the posterior aspect compared to three in the ≥15° varus group; this ambiguity could be due to associated FFD rather than genu varus. The removal of osteophytes from the posterior part of the femur was performed in 18 patients in the <15° group compared to 4 in the ≥15° varus group. Four patients in the <15° varus group had pie crusting of the MCL compared to two in the ≥15° varus group. None of the patients had required rectus sniff, nor had avulsion of the patellar tendon. The comparison of intraoperative flexion among the study participants is mentioned in Table 5.

**Table 5:** Comparison of intraoperative flexion among the study participants

Varus degree	Number (n)	Mean	Standard deviation	Standard error of mean	P-value
<15°	20	103.00	9.787	2.188	0.024
≥15°	47	92.34	19.528	2.849	

Three patients in total (one in varus <15°, two in varus >15°) required breakthrough analgesia for pain and received a single dose of IV tramadol (50 mg), twice on post-operative day 1. This adds up to 10 morphine milligram equivalents (MME) per day in each patient.

## Discussion

Achieving effective control of pain after TKA continues to be a significant clinical issue in facilitating the process of rehabilitation and enhancing the satisfaction of the patient. The main objective of this study was to evaluate the efficacy of pre-emptive analgesia with etoricoxib 60 mg and pregabalin 75 mg in patients undergoing TKA. The results confirmed the hypothesis that pre-emptive analgesia could alleviate post-operative pain reflected by VAS pain scores.

Gabapentin, an analog of aminobutyric acid, has been approved for the control and prevention of partial seizures. It has also been shown to relieve neuralgia that is postherpetic and to help in restless leg syndrome [13,14]. It is also responsible for reducing the activity of the receptors of the spinal cord and causes a reduction in hyperalgesia [14]. Gabapentin has been used as an analgesic, due to its inhibitory effects on the nociceptors, for ‘pre-emptive’ analgesia [15,16].

A significant decrease in the need for analgesics, including opioid agents, was documented by Lindberg-Larsen et al. when they advocated a pre-emptive medication on the TKA day, followed by its use for three consecutive days one hour before mobilization [17]. It facilitated effective rehabilitation in achieving adequate knee joint flexion. Pregabalin and gabapentin have been used as pre-emptive analgesics that can reduce the need for post-operative analgesic rescue effectively and delay the requirement of the analgesic. Gabapentin has been found to help in reducing the composition of opioids 24 hours post-operatively, but the effect does not depend on the dose. The doses that can be considered safe in consuming pregabalin and gabapentin are 300 and 1200 mg, respectively [18].

Every procedure of surgery, including TKA, breaks the vascular endothelium, increasing the vascular permeability, which can lead to edema and initiation of the inflammatory cascade at the site of operation. By affecting the activity of cyclooxygenase and lipoxygenase, methylprednisolone imposes restrictions on factors that stimulate nociceptors and directly affects the neuronal pathway of acute pain [19]. Multimodal analgesia after arthroplasty is not a part of current practice guidelines, and gabapentin application is controversial. In a study, gabapentin did not demonstrate a decrease in the post-operative pain and consumption of morphine. This study found that pregabalin, combined with etoricoxib, effectively reduced post-operative pain after 48 hours of surgery [20].

Wang et al. found that the pre-operative use of cyclooxygenase 2 (COX-2) selective inhibitors was associated with a significant reduction in knee pain and consumption of morphine after TKA [21]. The meta-analysis showed that compared with the control group, selective COX-2 inhibitors reduced the amount of morphine used 72 hours after surgery [22]. In an another study, pre-emptive analgesia with pregabalin and etoricoxib was initiated, and the post-operative pain was measured with the help of VAS. This measurement was done on the first and third days post-surgery. The VAS score showed a progressive decrease on each consecutive post-operative day with a statistically significant reduction observed in all three individual cohorts [21]. The usage of celecoxib 400 mg along with pregabalin 150 mg one hour prior to TKA as pre-emptive analgesia has also been shown to lower opioid consumption and reduce post-operative pain in other studies with results similar to this study [12,21].

The primary fact that the range of movement (ROM) gets affected directly by pain indicates that reducing the post-operative pain with pre-emptive analgesia increases the ROM and the early rate of mobilization in patients. This study focused on the improvement of ROM daily. Still, no such difference was observed in the ROM between the subjects who were provided pre-emptive analgesia as compared to the <15° varus group [22]. Pre-emptive analgesia is expected to permit mobilization in patients in the early post-operative stages. Early mobilization reduces several complications such as deep vein thrombosis and promotes the healing process, improving functional outcomes. In this study, we did not find differences that were significant in terms of the time required for mobilization between the two groups [23].

Buvanendran et al. found that of the 240 patients undergoing TKA, patients who received pregabalin 300 mg pre-operatively and 14 days after the process of surgery had reduced post-operative consumption of opioids and improved process of mobility during rehabilitation, with decreased scores of pain on passive ROM at discharge. To our knowledge, this is one of the first studies to correlate the extent of medial release in TKA with the VAS score and severity of varus deformity [24]. Our study showed that the release of the posterior capsule was associated with significantly higher VAS scores in patients with severe varus deformity >15° in whom it was routinely performed.

There are also some limitations to our study. A comparison of the outcome of pre-emptive analgesia was done between two groups of patients with OA knee with less than 15° and more than 15° genu varum, and there was a lack of a control group. The unequal sample size in both the groups could have given rise to confounding results. There was no documentation regarding the role of pre-emptive analgesia concerning the sex of the individual. Also, we were unable to assess post-operative mobility as the rate of mobilization was different in different patients.

## Conclusions

It was found that the pre-emptive usage of the combination of etoricoxib 60 mg and pregabalin 75 mg two hours before surgery helped in reducing the post-operative pain. It also reduced different intra-operative surgical maneuvers in patients undergoing TKA with different degrees of genu varus (less than 15° and more than 15°). The present study is an effort to highlight the usage of both the aforementioned drugs in order to achieve desired outcomes, especially in the post-operative period. Further large-scale studies would help in managing these patients and would help in modifying the treatment guidelines.
